# Honesty in signalling games is maintained by trade-offs rather than costs

**DOI:** 10.1186/s12915-022-01496-9

**Published:** 2023-01-08

**Authors:** Szabolcs Számadó, István Zachar, Dániel Czégel, Dustin J. Penn

**Affiliations:** 1 Department of Sociology and Communication, Budapest University of Technology and Economics, Egry J. u. 1, Budapest, H-1111 Hungary; 2 CSS-RECENS “Lendület” Research Group, MTA Centre for Social Science, Tóth Kálmán u. 4, Budapest, H-1097 Hungary; 3 Institute of Evolution, MTA Centre for Ecological Research, Konkoly-Thege Miklós út 29-33, Budapest, H-1121 Hungary; 4 Department of Plant Systematics, Ecology and Theoretical Biology, Biology Institute, ELTE University, Pázmány P. sétány 1/C, Budapest, 1117 Hungary; 5 Doctoral School of Biology, Institute of Biology, Eötvös Loránd University, Pázmány Péter sétány 1/C, Budapest, H-1117 Hungary; 6 BEYOND Center for Fundamental Concepts in Science, Arizona State University, AZ 85287–0506, Tempe, AZ USA; 7 Department of Interdisciplinary Life Sciences, Konrad Lorenz Institute of Ethology, University of Veterinary Medicine, Vienna, Savoynestrasse 1a, 1160 Vienna, Austria

**Keywords:** Animal communication, Honest signals, Handicap Principle, Costly signalling theory, Life-history models, Trade-offs, Evolutionary stability

## Abstract

**Background:**

Signal reliability poses a central problem for explaining the evolution of communication. According to Zahavi’s Handicap Principle, signals are honest only if they are costly at the evolutionary equilibrium; otherwise, deception becomes common and communication breaks down. Theoretical signalling games have proved to be useful for understanding the logic of signalling interactions. Theoretical evaluations of the Handicap Principle are difficult, however, because finding the equilibrium cost function in such signalling games is notoriously complicated. Here, we provide a general solution to this problem and show how cost functions can be calculated for any arbitrary, pairwise asymmetric signalling game at the evolutionary equilibrium.

**Results:**

Our model clarifies the relationship between signalling costs at equilibrium and the conditions for reliable signalling. It shows that these two terms are independent in both additive and multiplicative models, and that the cost of signalling at honest equilibrium has no effect on the stability of communication. Moreover, it demonstrates that honest signals at the equilibrium can have any cost value, even negative, being beneficial for the signaller independently of the receiver’s response at equilibrium and without requiring further constraints. Our results are general and we show how they apply to seminal signalling models, including Grafen’s model of sexual selection and Godfray’s model of parent-offspring communication.

**Conclusions:**

Our results refute the claim that signals must be costly at the evolutionary equilibrium to be reliable, as predicted by the Handicap Principle and so-called ‘costly signalling’ theory. Thus, our results raise serious concerns about the handicap paradigm. We argue that the evolution of reliable signalling is better understood within a Darwinian life-history framework, and that the conditions for honest signalling are more clearly stated and understood by evaluating their trade-offs rather than their costs per se. We discuss potential shortcomings of equilibrium models and we provide testable predictions to help advance the field and establish a better explanation for honest signals. Last but not least, our results highlight why signals are expected to be efficient rather than wasteful.

**Supplementary Information:**

The online version contains supplementary material available at 10.1186/s12915-022-01496-9.

## Significance statement

Honest signals pose a major theoretical problem for understanding animal communication, as it is unclear what prevents deception. The leading explanation for honest signals has long been the Handicap Principle, which predicts that signals are honest if are costly to produce, as it is their costliness (or wastefulness) that prevents deception and the breakdown of communication. However, in the models that reportedly validated the Handicap Principle (e.g. [1, 2]), the costs of signalling are derived from a set of rather specific assumptions, restricting the possible outcomes. These results were over-generalized and it was mistakenly concluded that honest signals must always be costly at the evolutionary equilibrium. Therefore, models are needed to investigate how signalling costs — better labelled signalling tradeoffs — influence the evolution of honesty without unnecessarily restrictive assumptions. Here, we provide a mathematical model based on general assumptions about signalling trade-offs that show how the evolution of cost-free or even beneficial honest signals can be evolutionary stable at equilibrium. Our results show that honest signals need not be costly at all, and therefore, the Handicap Principle and other costly signalling models cannot provide a general explanation for understanding the evolution honest signals. Our model provides a more general approach for addressing the evolution of honesty and deception in animal communication. We give testable predictions to advance the field and our model demonstrates why the handicap paradigm should be abandoned.

## Background

Explaining the evolution of honest signalling has been a long-standing problem in research on animal [3] and human communication [4]. Zahavi’s Handicap Principle [5, 6] (HP) has been the leading theoretical paradigm for honest signalling since it was reportedly validated by Grafen’s ‘strategic handicap’ model [1]. The HP predicts that signals must be costly and reduce survival at the evolutionary equilibrium — hence the label ‘handicap’ — in order to be honest. This idea is often claimed to provide a general principle to explain why signals are honest, and it has been widely accepted, although some have questioned its generality [7]. There is no consensus for how to define, model or test the HP, which is often confused with other models known as ‘costly signalling theory’, because handicaps and signalling costs have never been clearly defined, and it has never been shown how signalling costs *per se* enforce honesty [7–10].

Mathematical signalling games have greatly improved our understanding of honest signalling [3, 11, 12], as they have clarified the logic of honesty in conspecific interactions, including aggression [13], mate choice [1], parent-offspring conflict [2, 14, 15], and interspecific interactions, such as plant-herbivore [16], plant-pollinator [17], aposematic displays [18], and predator-prey [19] relations. They originated from economic signalling games [20] and have been used to analyse the stability of honest signals in a variety of human social interactions [21]. While these models have proved to be useful, identifying the costs of signalling at the honest evolutionary equilibrium (equilibrium cost function) in such models is far from trivial when the signallers’ quality varies continuously [1, 2]. As a consequence, it is difficult to compare the outcome of these models and make any general conclusions.

The so-called ‘strategic handicap’ model [1] is the most influential model of honest signalling, and it critically assumes that signallers differ in their quality and bear differential marginal costs for producing a signal. This is a plausible but widely misinterpreted model because it is very different from the HP. [7] Unlike the HP, honesty in this model does not depend upon the absolute costs of signalling and signals are efficient rather than wasteful. Moreover, honest signals are selectively favoured in the model despite of their costs; not because they are costly. This model has nevertheless provided an important step towards analysing fitness trade-offs for honest signalling, but the steps used to obtain the equilibrium cost function are difficult to replicate, hence the mathematics have been described as ‘brilliant but arcane’ [9].

The complexity of signalling games has been widely under-estimated, as it has been generally overlooked that finding an equilibrium cost function requires solving a double optimization problem [14, 22], one for the receiver as well as one for the signaller, and that the optimal solution for the signaller depends on the receiver’s optimum. This complexity is daunting and it has been circumvented by ignoring the receiver’s optimization problem (Additonal file 1: sections 1–4 gives a detailed description of the steps to solve this problem; while Additional file 1: sections 5–7 provides a more detailed discussion [1, 2, 14, 22–30];). This issue cannot be resolved until the optimization problem of the signaller and the receiver are both evaluated.

This double optimization problem has an infinite number of possible solutions [22, 31], and no general solution has ever been provided in analytical form. The lack of a clear methodology for deriving solutions to address this double optimization problem contributes to widespread misinterpretations of the HP and Grafen’s strategic choice model (see [7]) and left the critiques of these ideas difficult to understand [8, 9] and the debates unresolved. It has been known for 20 years that such signalling games have an infinite number of honest equilibria [22, 31], and yet the nature and implications of these equilibria have remained unexplored due to the complexity of this problem. Consequently, the conditions for honest communication in signalling games are still unclear and controversial, and the field has stagnated due to being entwined in the erroneous and confusing handicap paradigm [7].

Here, we provide a novel and general approach for determining stable equilibria in continuous signalling games, and for calculating equilibrium signal cost functions, as a continuation of previous theoretical developments [23]. We examine signalling models with *additive fitness functions* (when signal costs and benefits are measured in the same currency, such as fitness), and also *multiplicative fitness functions* (such as when signals have survival costs that influence their reproductive benefits) [32]. First, we describe an asymmetric signalling model of animal communication, and we aim for a general approach that will apply to any signalling context, given that certain, broad conditions are met. Then, we provide solutions for games with additive or multiplicative fitness functions. We provide a formal proof of the conditions for stability being independent of equilibrium signal cost. Our general formula specifies the full, infinite set of trade-off solutions of the double optimization problem. Furthermore, we show that an infinite number of cost-free and negative cost equilibria exist in these models. The discovery of these previously unknown and evolutionarily stable equilibria shows how new approaches and interpretations can be used to investigate signalling games in general.

Our approach does not require prior knowledge or assumptions about the shape of the potential solution, and hence it is applicable to any signalling model. We apply our method to calculate stable equilibria in classic signalling models, including Grafen’s model of sexual signals [1], Godfray’s signal-of-need model [2] for parent-offspring signalling games, and for the signalling model of Bergstrom et al. [22]. We explain how our results provide testable predictions regarding cost-free and beneficial (negative-cost) honest signals at equilibrium, and how these could support (or refute) our results and their generality. Finally, we discuss the shortcomings of equilibrium models and how signalling theory fits into the larger framework of life-history theory and Darwinian evolution.

### Models of signalling games

**Fig. 1:**
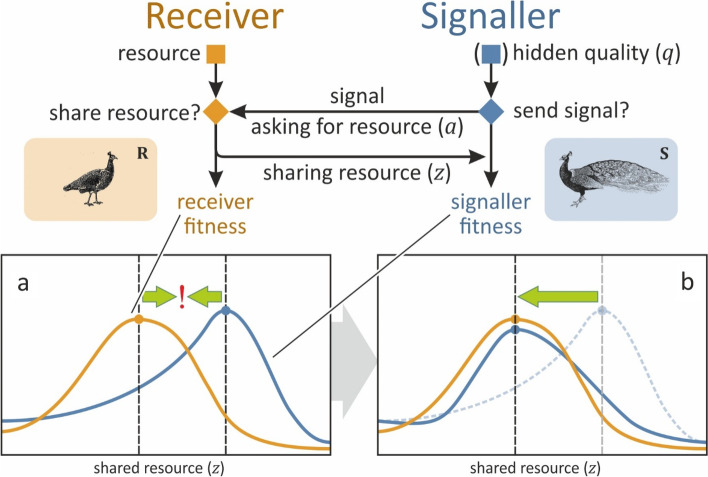
Female mate choice: an asymmetric signalling game. Courtship and mating behaviour is a sexual interaction in which a male signaller (S) expresses a signal, a secondary sexual trait, that functions to persuade choosey female receivers (R) to mate and give him on opportunity to fertilize her eggs; a scarce resource (e.g. [1]). Males vary in their quality and R will mate with S depending upon his quality or condition. S invests into producing a signal, according to his quality (he need not consciously ‘know’ his own quality; such a mechanism only requires condition-dependence), whereas R cannot evaluate his quality directly; she must decide based solely on attributes of S’s signal (i.e. there is an information asymmetry between S and R). Axes *x* and *y* in the inset images represent the amount of shared resource *z* and fitness w outcomes respectively. Inset **a**: For a given signaller quality and no signalling trade-off, the fitness curves of S (blue) and R (yellow) have their optima (blue and yellow points) at different amounts of shared resource (dashed lines), resulting in a conflict of interest. Inset **b**: Signal trade-offs modify the signaller’s fitness curve (blue) such that its optimum is at the same amount of resource that the receiver is willing to share, reducing or eliminating the conflict in the interaction. Costly signalling theory predicts that this trade-off function must be positive at the equilibrium for signalling to be honest [1, 2]

Signalling games are mathematical models used to analyse how individuals (*signallers)* attempt to influence the decisions of others (*receivers)* by producing signals (action at a distance, see Fig. 1). *Signals* are often strictly defined as traits that provide information about some aspect of a signaller not directly observable, such as size or sex; otherwise signals are unnecessary [20]. Signalling games are usually described as conflicts over a resource, because some of the first models were contests over food and territories. Indeed, from an evolutionary perspective, a receiver’s body and behaviour can be viewed as resources over which signallers compete to exploit for their own benefit [33]. Signalling games can be symmetric or asymmetric concerning information, resources, and options (strategies) available to the players. In symmetric games, players have the same information sets, resource availability and strategies at the beginning of the game, whereas in asymmetric games, players do not share the same information, resources, or strategic options.

Games can be symmetrical or asymmetrical in many respects, though it was information asymmetries between signallers and receivers that have mainly attracted the interest of biologists [34] and economists [20, 35]. Asymmetrical information is common in nature and thus asymmetrical signalling games have often been used to investigate how individuals resolve a wide variety of interactions and types of *conflicts* (e.g. genomic, sexual, parent-offspring, and other intra- and inter-familial conflicts). In these models, signallers use signals to persuade a receiver to take some action, which can include mating [1], feeding [2], other forms of parental investment [36], committing suicide [37, 38], and performing other actions that may or may not be in the receiver’s interest. Asymmetrical signalling games have been used to model intra-genomic conflicts and molecular signals between cells within the body [39, 40]. They have also been used to model a variety of interspecific interactions, including predator-prey [19, 41], host-parasite [42–44], plant-herbivore [16], plant-pollinator [17] and aposematic displays [18]. They are also used to understand and address the spread of misinformation and disinformation in human societies, which is arguably one of the most important problems facing our species [45–47].

Here, we focus on games with asymmetries in access to both information and resources. In asymmetric games, receivers possess a resource and can decide whether to share it with signallers or not. For example, young chicks attempt to persuade their parents to feed them by producing begging calls [2]. In discrete models, receivers can either give away the entire resource or keep it for themselves [11, 16, 19, 48–50], whereas in continuous models, receivers can share some *portion of the resource* (*z*) [1, 2, 14, 22, 23, 31, 32, 51]. Receivers are assumed to share the resource in a way that maximizes (inclusive) *receiver fitness* (*w*_**R**_), but the potential benefits depend upon obtaining reliable information from signallers about what they offer in exchange. The problem is that receivers often have incomplete information about signallers or what they have to offer (information asymmetry). In the case of mate choice, females assess the potential benefits of mating with males that differ in social status, health, resources, or other aspects of *quality* (*q*); however, male quality cannot be directly assessed by the receiver, otherwise there is no need for signals. The signaller can influence the receiver’s decision by its signal, which may or may not reliably reveal the quality *q* of the signaller, to *ask for the resource amount* (*a*) that should maximize *signaller fitness* (*w*_**S**_) (see Fig. 1). A signal is ‘honest’ if it provides receivers with reliable information about the signaller’s quality, allowing the receiver to make adaptive decisions. Alternatively, the signal can be useless or deceptive, so that signallers manipulate the receivers to share more than an amount *z* that is in their adaptive interest. Like previous honest signalling models, we investigate the conditions under which signals provide reliable indicators of quality *q* (for details, see the ‘Methods’ section and Additional file 1:sections 1–3).

Theoretical models have previously shown that honest signals are evolutionarily stable at an *honest equilibrium* if the following conditions are met [14, 22]: (*i*) the signal reveals the signaller’s actual quality *q* (signals are honest), so that the receiver can respond adaptively; or (*ii*) the signaller only asks for the amount *a* of a resource that receivers benefit by sharing (shared interest), so that the conflict between the receiver and signaller is removed at the honest equilibrium (*a* = *z*). The mathematical formulation of these conditions is detailed in the ‘Methods’ section.

The standard theoretical approach used for resolving conflicts of interest and to find stable equilibria in asymmetric signalling games is to introduce a *cost function* that transforms the signaller’s fitness function *w*_**S**_, so that the optimal amount of resource *a* acquired by the signaller corresponds to the optimal amount of resource that the receiver shares *z* (see Fig. 1), and the optima of signaller and receiver, namely, *w*_**S**_ and *w*_**R**_, then coincide. This step is crucial but missing from many previous models. It is not enough to find the optimum of *w*_**S**_, but *w*_**S**_ must be transformed by using a function traditionally referred to as a ‘cost function’ in which max(*w*_**S**_) = max(*w*_**R**_). Here, we will refer to this transformation as a trade-off function (*T*). The function *T* transforms the benefit into the actual fitness so it is actually a trade-off function. Accordingly, the signallers’ fitness *w*_**S**_ is determined by the relation between the benefits *B* and trade-offs *T*, and without any trade-offs, *w*_**S**_ = *B*. In additive models (e.g. [2]), *B* and *T* are summed, whereas in multiplicative models (e.g. [1]), they are multiplied to yield the fitness *w*_**S**_.

We use the term ‘trade-off function’ because the term ‘cost function’ is unnecessarily restrictive to the positive domain (counter-intuitively, the cost value is positive rather than negative), and moreover, it does not represent the full set of possible solutions, as we demonstrate below. We also avoid the term ‘cost function’ because it has generated much confusion, and we provide a more detailed explanation in the ‘Discussion’ section. Our key insight is that this transformation, regardless of its label, does not necessarily represent an absolute cost, whereas it is always defined by a trade-off *sensu* life-history theory.

We construct the most general class of trade-off functions that obey the conditions of honest signalling for both additive and multiplicative fitness functions (see the ‘Methods’ section and Appendices 2–3). Lastly, we apply our method to well-known models of honest signalling (Appendix 4), demonstrating its general applicability.

## Results

**Fig. 2:**
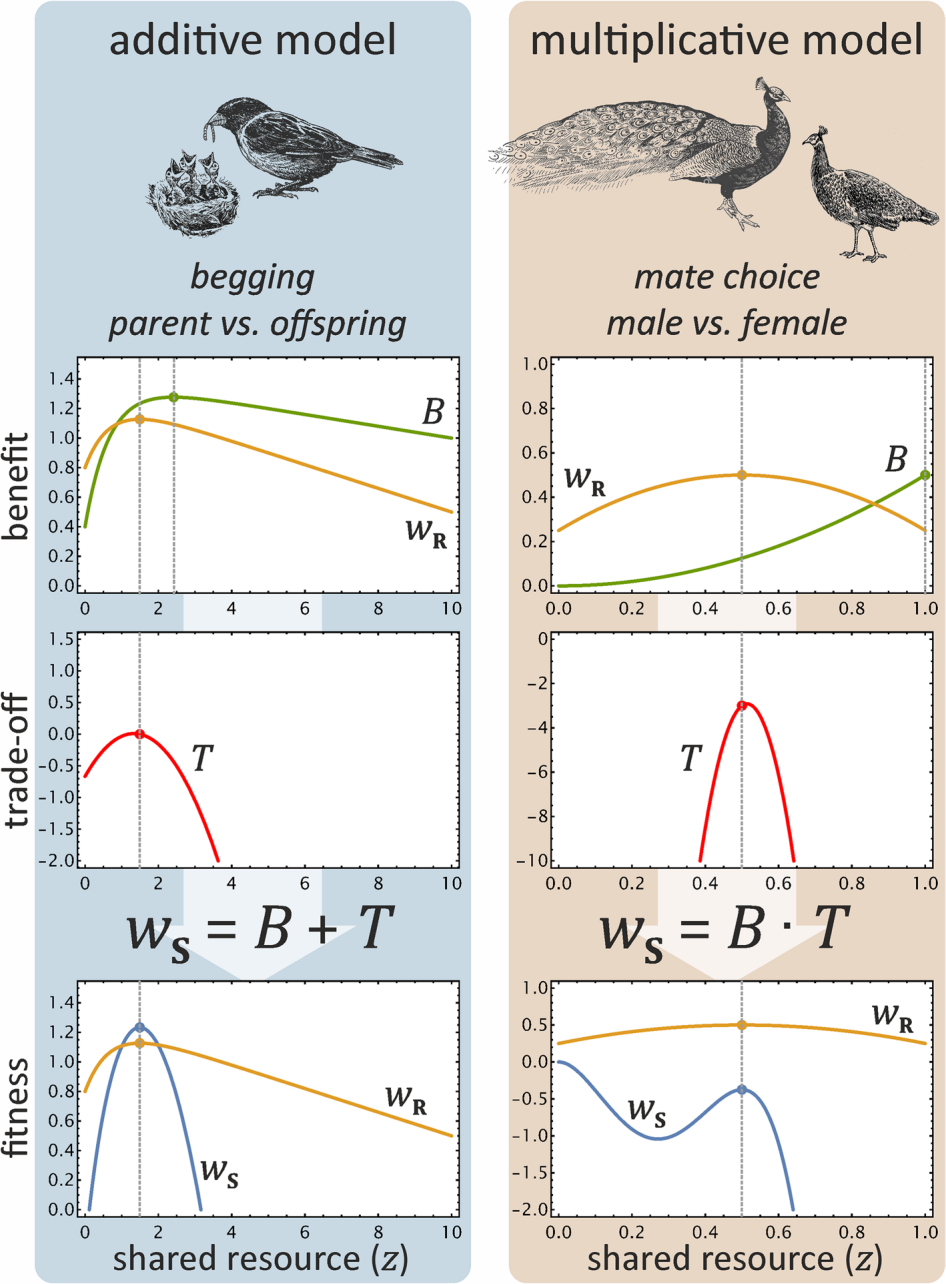
Cost/benefit trade-off functions for two traditional signalling games. Left: An additive offspring begging game. Right: A multiplicative mate choice signalling game. Dashed vertical lines indicate the receiver’s and the signaller’s optimal amount of resource, given a signaller’s quality or condition. Trade-off function *T*, when added to or multiplied by *B*, transforms the signaller’s benefit *B* to its actual fitness *w*_**S**_ such that its optimum amount of requested resource *a* coincides with the amount *z* shared by the receiver

**Fig. 3:**
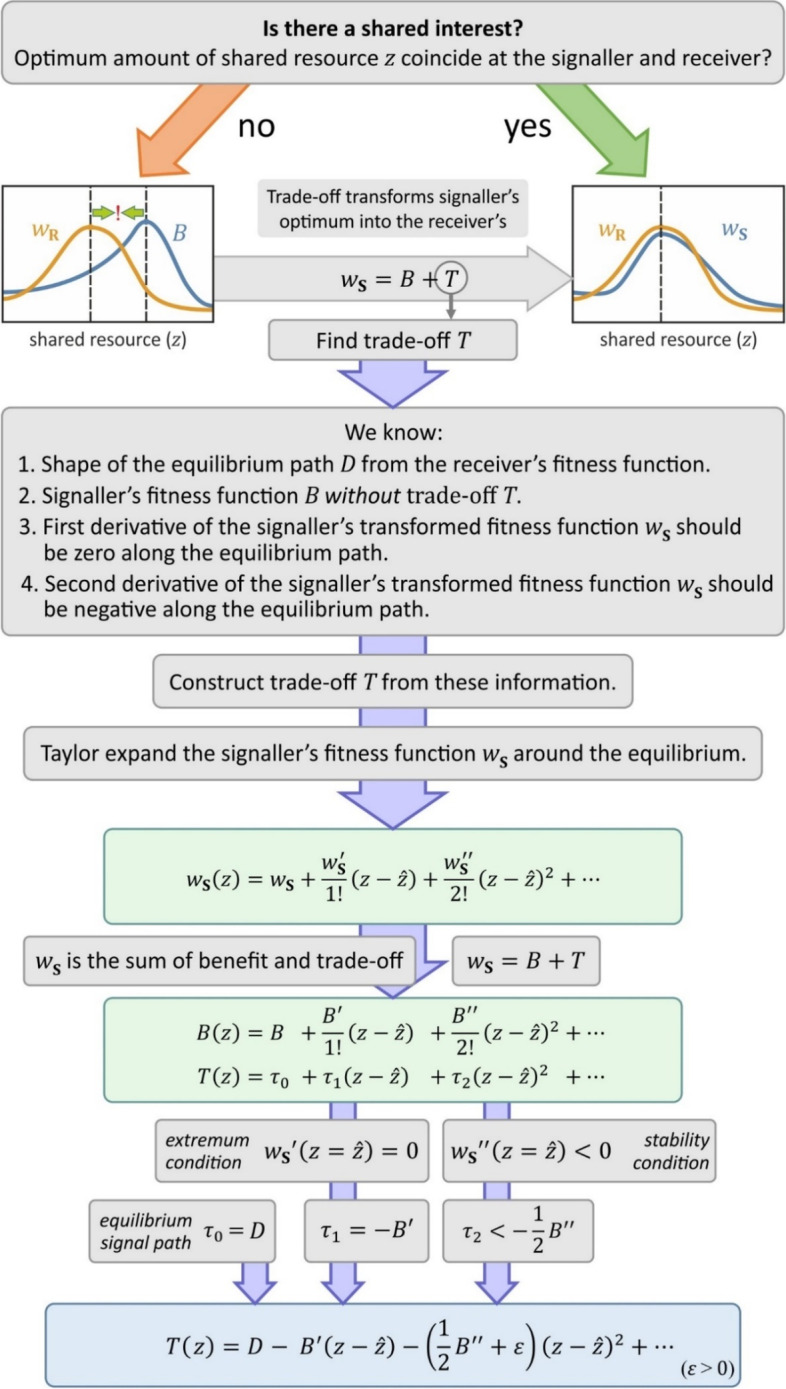
Method for reverse-engineering the general trade-off function *T*. The method transforms any (at least twice) differentiable signaller benefit function *B* to the fitness function *w*_**S**_ that has the same optimal amount of shared resource as the receiver’s fitness function *w*_R_. For sake of simplicity, function arguments are omitted at the right side of equations. *D* and higher order Taylor coefficients *τ*_3_, *τ*_4_, … can be freely chosen

Since it is the fitness *w*_**S**_ that must meet the conditions of stability and honesty in an honest equilibrium, and not the benefit *B*, we first show that a signal trade-off function *T* can always be found for any *B* that ensures that *w*_**S**_ meets these conditions. In order to decompose the signaller’s fitness function into terms that are in one-to-one correspondence with the conditions of honest signalling, we expand the signaller’s fitness function to its Taylor-series around (honest) signalling equilibrium. This representation allows us the derive the exact and most general implications of the conditions of honest signalling term by term. As we show below, the conditions of honest signalling constrain the first order and second order terms, while the rest can be chosen arbitrarily. When terms are again summed up, the resulting *w*_**S**_ represents all honest solutions of signalling. Fig. 2 illustrates the process for additive and multiplicative models, while Fig. 3 provides a visual guide for the method of constructing *T* for the additive case (see the ‘Methods’ section, Appendix 2 and Fig. S1).

### Additive fitness

The general form of any (at least twice differentiable) trade-off function for additive fitness *T*_*A*_ using Taylor-series expansion around the equilibrium where $a=z=\hat{z}$ is (see Appendix 2 and Fig. S1 for details):

$${T}_A\left(q,z\right)=D(q)-{B}^{\prime}\left(\hat{z}\right)\cdot \left(z-\hat{z}\right)-\left(\frac{1}{2}{B}^{\prime \prime}\left(\hat{z}\right)+\varepsilon \right)\cdot {\left(z-\hat{z}\right)}^2+\dots .$$

**Fig. 4:**
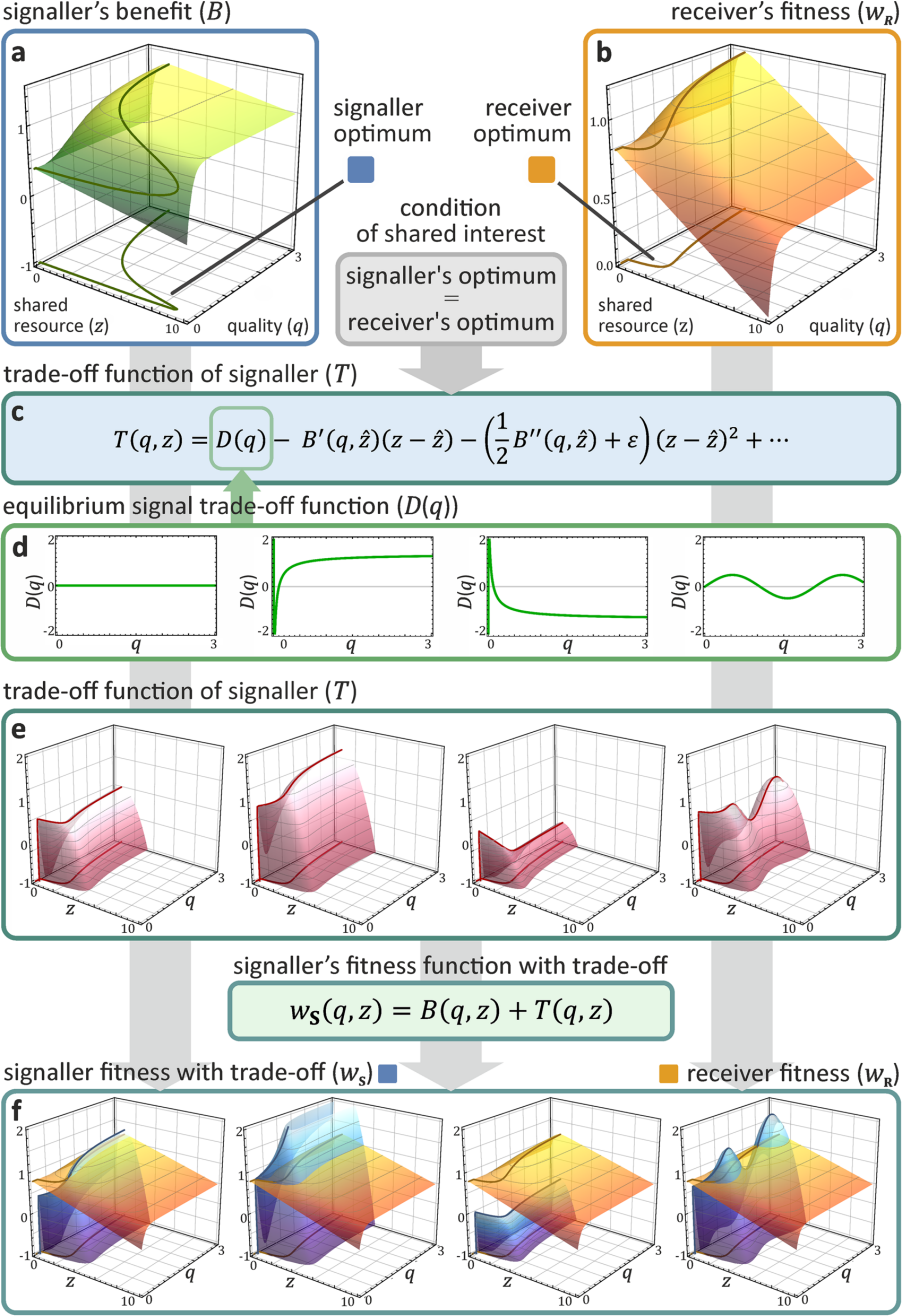
The effect of different trade-off functions on the fitness of the signaller in case of Godfray’s additive model of parent-offspring conflict [15]. **a** The signaller’s benefit function *B* (without trade-off; dependent on its quality *q* and the received amount of resource *z*) defines its optimum strategy for any *q* (dark green curve; optimum curves are also projected onto the *q* − *z* baseplane for all surfaces). **b** The receiver’s fitness function *w*_**R**_ defines its optimum strategy for any signaller quality and resource shared (yellow curve). **c** At the honest equilibrium, the trade-off function *T* ensures that the signaller’s optimum coincides with the receiver’s optimum (for the derivation of the terms of *T*, see Fig. 3). **d** An arbitrary set of equilibrium signal trade-off functions *D*(*q*) is selected (green curves) from left to right: $\left\{{D}_1(q)=0,{D}_2(q)=B\left(q,\hat{z}\right),{D}_3(q)=-B\left(q,\hat{z}\right),{D}_4(q)=\sin (3q)/2\right\}$, where $\hat{z}$ is the optimum transfer of the receiver for the given quality *q*. **e** For any *D*_*i*_(*q*), a trade-off function *T*_*i*_ is generated (red surfaces), describing the cost value of signals in and out of equilibrium. **f** The trade-off function *T* transforms the benefit function *B* of the signaller to the fitness function *w*_**S**_ (blue surfaces) such that its optimum strategy coincides with the receiver’s optimum strategy (yellow surfaces replicate the receiver’s fitness *w*_**R**_ as of panel **b**; note different scaling). Projected optima of *w*_**S**_ and *w*_**R**_ entirely overlap at the *q* − *z* baseplane. Parameters are {*ψ* = 1/2, *γ* = 1/2, *G* = 0.08, *U* = 1, *Z* = 10, *ε* = 1}, for details, see Appendix 2 and 4

The zero order Taylor coefficient *D*(*q*) is the *equilibrium signal trade-off function* of signaller quality *q*. Traditionally, this coefficient has been used to specify the cost that signallers pay at the equilibrium, independently of the conditions of honest signalling [2]. Fig. 4d shows examples of costly and cost-free equilibrium trade-off functions.

The second coefficient $-{B}^{\prime}\left(\hat{z}\right)$ describes the *equilibrium path,* where the first derivative of *w*_**S**_ with regard to the amount of shared resource *z* is zero. This coefficient specifies that ${w}_{\textbf{S}}\left(\hat{z}\right)$ is an extremum, according to the shared-interest condition (${w}_{\textbf{S}}^{\prime }=0$, Eq. 1a). At the equilibrium max(*w*_**S**_) = max(*w*_**R**_), and thus this path represents the receiver’s optimum as well.

The third coefficient $-\left(\frac{1}{2}{B}^{\prime \prime}\left(\hat{z}\right)+\varepsilon \right)$ determines the steepness of the surface along the *z* dimension when deviating from the equilibrium path (*stability condition*). The condition *ε* > 0 ensures that this term is larger than the second derivative of *B* for the slope to be negative (${w}_{\textbf{S}}^{\prime \prime }<0$, see Eq. 1b) so that ${w}_{\textbf{S}}\left(\hat{z}\right)$ is a maximum. When this term is equal or smaller than the second derivative of *B* then the signaller’s strategy is not an equilibrium strategy. The conditions of honest signalling do not restrict higher order terms of the series therefore they can be arbitrarily chosen.

The Taylor series expansion allows functional decomposition of the equilibrium trade-off, equilibrium path, and stability. Accordingly, the equilibrium trade-off function *D*(*q*) can be negative, zero or even positive. These conditions can be interpreted as costly signals, cost-free signals, and signals with only benefits, respectively. For any equilibrium path, (i.e. second coefficient), reflecting the receiver’s optimisation problem, there is an infinite number of equilibrium trade-off functions *D*(*q*) for the signaller (see Fig. 4d for examples). In general, the *equilibrium* trade-off function (zero order term) is not constrained by the *equilibrium path* (first order term) or the *stability condition* (second order term).

Figure 4d shows four possible equilibrium signal trade-off functions with constant, monotonically increasing, monotonically decreasing, and oscillating trade-off functions. While the last choice seems unrealistic, it proves our point that any arbitrary, continuously differentiable function can be chosen as the equilibrium trade-off function *D*(*q*) because *the equilibrium signal cost is independent of the stability condition*.

### Multiplicative fitness

For multiplicative fitness, the conditions of honest signalling imply the following general form for the trade-off function *T*_*M*_ (derivatives are all evaluated at equilibrium $z=\hat{z}$; see Additional file: section 3 and Additional file 1: Fig. S1 for details):

$${T}_M\left(q,z\right)=D(q)-\frac{B^{\prime }D(q)}{B}\left(z-\hat{z}\right)-\left(\frac{D(q)\ }{B}\left(\frac{B^{\prime \prime }}{2}-\frac{{\left({B}^{\prime}\right)}^2}{B}\right)+\varepsilon \right){\left(z-\hat{z}\right)}^2+\dots .$$

While the same functional separation is derived as in the case of additive fitness functions, the same independence of terms cannot be achieved because of the multiplication of the functions. Previous models have shown that signal cost functions *D*(*q*) exist where the cost paid at the equilibrium by honest signallers is arbitrarily close to zero in multiplicative models (e.g. this result was derived in a previous signalling model for a specific cost functions [31] from another signalling model [1]). Our formula for *T*_*M*_ provides a method to derive all solutions for any asymmetrical signalling game with continuous (and at least twice differentiable) fitness functions. Moreover, as a novel result, it reveals, that *equilibria with cost-free or beneficial signals exist in multiplicative models too*, not only in additive models.

In Additional file 1: section 4, we derive the general trade-off functions for well-known biological signalling games [1, 2, 22]. Additional file 1: Table S1 provides a comparison of notation across these models, Additional file 1: Table S2 compares the Taylor coefficients of the general trade-off functions of the additive and multiplicative cases, while Additional file 1: Table S3 compares the Taylor coefficients of relevant models.

## Discussion

Our methods and results provide several important contributions towards understanding the evolution of honest signals. First, we provide a general methodology for deriving the full set of an infinite number of trade-off functions for asymmetric, continuous pairwise signalling games, which allow for honest signalling. This general class of trade-off functions consists of three components: the first term defines the cost (or benefit) of a signal at the evolutionary equilibrium, the second one defines the path along the equilibrium, and the third term specifies the stability condition at the equilibrium. Second, we confirm the suggestion that the results of asymmetric signalling models depend upon whether fitness effects are multiplicative or not [9]. For additive fitness, these three components are independent of each other, whereas for multiplicative fitness models, they are not. However, the equilibrium cost of signals can be anything, zero or even negative, in both types of models and yet signalling remains honest and evolutionarily stable as long as the stability condition is fulfilled. A negative cost implies that the signal is beneficial independent of the receiver’s response. We show the existence of such beneficial equilibria for seminal models of the field, including Grafen’s model of sexual selection [1] and Godfray’s model of parent-offspring communication [2] (see Appendix 4a,b). Third, these results show that signal costs at equilibrium are not a necessary condition for the evolution of honest signalling, contrary to Zahavi’s Handicap Principle [5] and handicap interpretations of Grafen’s theoretical model [1, 2]).

Our results reveal an important limitation and a surprising implication of simple asymmetric signalling games. Our model does not specify the magnitude of signal intensity at equilibrium, and just like the equilibrium signal cost, the magnitude can be any continuously differentiable function [17]. For example, in Godfray’s signalling model [2], the equilibrium signal intensity (as a function of quality *c*) has a maximum (see Fig. 2 at [14]). Accordingly, the quality half-space below *c* = 0.5 was omitted to ignore the maximum, resulting in a monotonic decreasing signal intensity function. More generally, it was recently shown that it is possible to construct such ‘dishonest’, non-monotonous functions for a large class of signalling games [17]. In summary, overly simplified game-theoretical models have generated the apparent paradox that honest signalling games, which assume honesty at equilibrium, need not result in honest signalling, i.e. they will not necessarily generate a monotone increasing or decreasing signal intensity function at the equilibrium. This paradoxical result implies that the simplest possible model of honest signalling has not been sufficiently constrained in previous models, as ‘honest’ solutions were allowed where the signal cannot be used to predict the actual quality of the signaller.

**Fig. 5:**
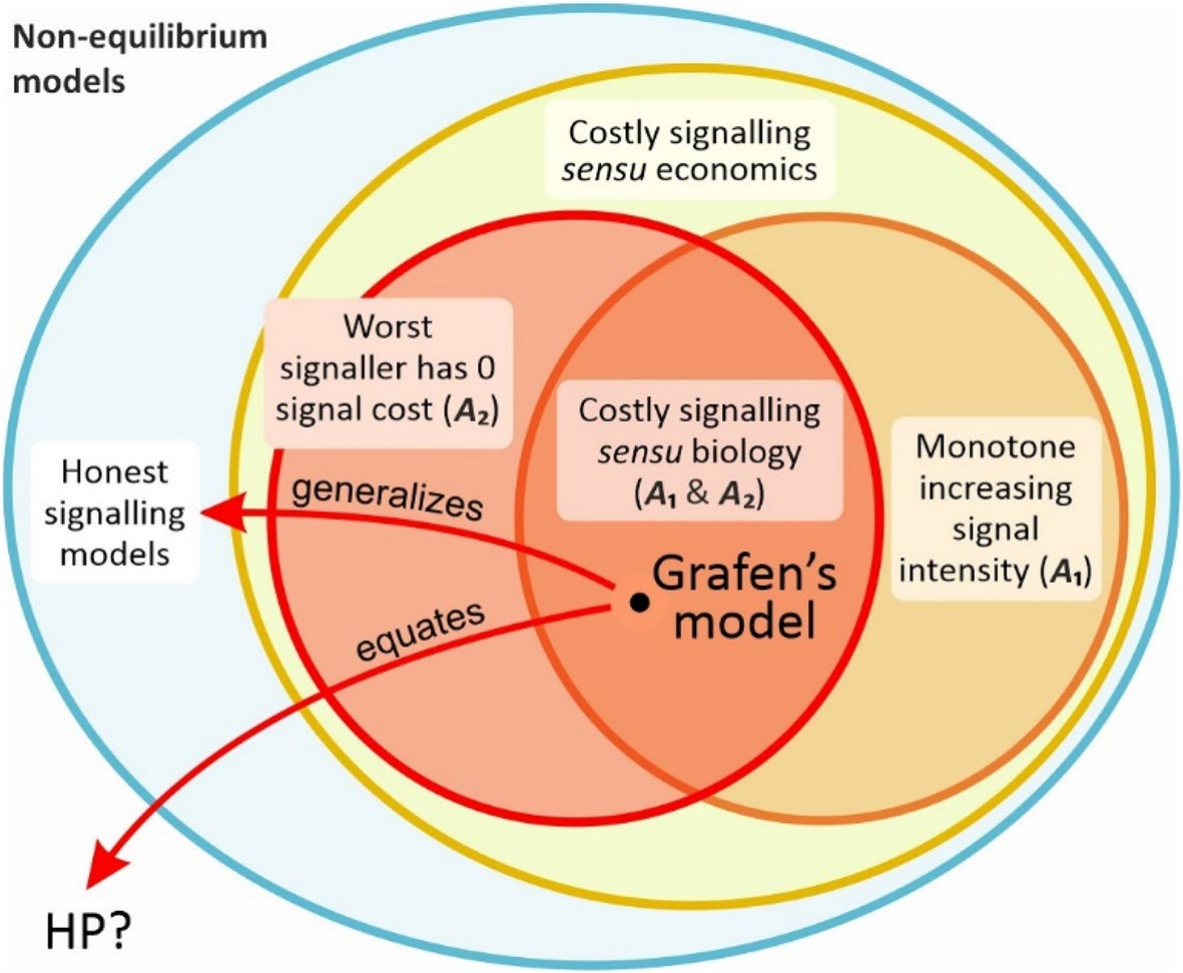
The overgeneralization fallacy and category mistake of Grafen’s model. The figure shows the relation between the potential set of honest signalling equilibria maintained by condition-dependent trade-offs (blue set) vs. ‘costly signalling’ *sensu* economics (yellow set) vs ‘costly signalling’ *sensu* biology (orange set). (i) When additional postulates are included that unnecessarily constrain a model, a conclusion may be correct, not because of the model, but because of the additional assumptions. When one nevertheless claims that the conclusion is generally true regardless of postulated assumptions, then this is an overgeneralization fallacy. (ii) If removing the constraints switches the conclusion (strongly depending thus on the assumptions), one has also committed a category mistake, incorrectly attributing properties to the model and missing its true nature. (iii) Standard costly signalling assumptions (SCSA = ***A***_1_ and ***A***_2_, orange set) unnecessarily constrain the model of honest signalling (yellow set), because they exclude a potentially important class of trade-off functions. Nevertheless Grafen overgeneralized the conclusion of his model to all signals of quality (red arrow to blue set; see overgeneralization fallacy, point (i)). (iv) Moreover, biologically relevant assumptions may not be constrained to SCSA , contrary to what Grafen suggested [1] (see the ‘Discussion’ section). Removing SCSA switches the conclusion ***C*** of the model to !***C***: honest signals need not be costly, as we have proved in this paper. That is, the conclusion of Grafen’s model (***C***= honest signals are costly), stems from its specific assumptions and not from more general properties, leading to tautology and a category mistake (see point (ii)) when Grafen identified his model with the Handicap Principle (red arrow to HP). That is, Grafen’s model is not a model of HP as the HP conclusions do not follow from the general properties of the model (it is a model of condition-dependent signals). Thus, even if honest signals turn out to be costly, the Handicap Principle cannot account for them

Existing models have other limitations, first introduced as biologically-inspired constraints [1, 2, 14, 22], and their most common assumptions are (*i*) the signal cost as a function of signal intensity increases monotonically (see above); and (*ii*) the equilibrium cost function *D*(*q*) is restricted by the assumption that the lowest quality signallers produce no signals and have no signal costs [1, 2, 14, 22]. The first assumption directly excludes any non-monotonic cost function (e.g. multimodal curves). The second assumption combined with the first excludes any potential cost function with zero or negative equilibrium signal cost. We call these the *standard costly signalling model assumptions*. When these assumptions are applied, the result is the traditional ‘costly signalling’ outcome in which the equilibrium cost function has positive values with monotonically increasing signal cost (i.e. equilibrium signals are honest and costly, e.g. see the strategic choice signalling model [1]). In other words, Grafen’s claim that signals must be costly (and wasteful) at the honest equilibrium does not follow from the most general formulation of the conditions of honest signalling; it follows only from the additional constraints — the *standard costly signalling model assumptions*. Note that the problem here is not the application of specific assumptions, but rather the misinterpretation that signal costs directly follow from the general formulation of the model (e.g. see the claim of Grafen ‘If we see a character which does signal quality, then it must be a handicap’ [1] p. 521). These assumptions may or may not be realistic, but the interpretation that a costly equilibrium is necessary for honesty is incorrect and does not follow from the general formulation (as signals in Grafen’s model are not honest because they are costly at the equilibrium). This misinterpretation of Grafen’s strategic handicap model (i.e. category mistake, see Fig. 5) led to the widespread acceptance of the HP and the popularity of costly signalling theory (for a detailed discussion of misinterpretations, see [7]). Grafen also made the mistake of overgeneralizing his ‘main handicap results’ to the full set of potential solutions (i.e. overgeneralization fallacy, see Fig. 5) when he claimed that all signals of quality must be handicaps (as honest signals need not be handicaps).

It is important to note that our findings also highlight the limitations of studying the evolutionary equilibrium for honesty using game theoretical models. Our formula clears up confusion over differences between conclusions that follow from the *conditions* of honest signalling models versus the consequences from *additional constraints*. We show that honest signalling models can only predict the value of marginal change – the behaviour of the system — in vicinity of the evolutionary equilibrium (by definition) without including additional constraints. They cannot provide predictions about (*i*) the cost of signals at or outside of the equilibrium, or (*ii*) the marginal change further away from the equilibrium path (see Fig. 4). One can add additional constraints to the models (see above discussion, e.g. [1, 2]) but then the results are simply determined by these constraints. Making such general conclusions from a model and ignoring its additional restrictive assumptions is an example of the *overgeneralization fallacy*. We have demonstrated here how removing the constraints of these previous models undermines their usual interpretations: honest signals need not be costly (see Fig. 5) even under conflict of interest, and *hence the HP can be fully rejected*.

Equilibrium models have another limitation: by definition, they investigate honest equilibria only. Whether other equilibria exist or not is out of the scope of our analyses. The argument that if honest equilibria exist, then all signals need to be honest (see Grafen’s ‘main handicap results’ [1], p. 521) is a *non sequitur*, as the existence of (partially) dishonest equilibria cannot be excluded. There is no guarantee that an honest equilibrium will eventually evolve as signalers and receivers may settle at an entirely or partially dishonest equilibrium. Equilibrium models cannot account for the dynamics leading to the equilibrium; for that, additional mechanisms are required, e.g. replicator dynamics [52] or individual-based simulations [53].

The results of our model help to explain why empirical studies do not match the predictions of the HP or costly signalling theory. Numerous studies have failed to find ‘costly signals’ predicted by Zahavi and Grafen (e.g. see [54–61]; for example, offspring begging calls are nowhere as costly as often assumed [54], see [55] for review). Not even the elaborate peacock’s train, the flagship example of the HP, fits the predictions of costly signalling models. The train does not hinder movement [56]; on the contrary, males with longer trains are able to take-off faster than males with shorter ones [57]. Empirical studies have often been cautious with their conclusions and suggested that some other types of signalling costs might be discovered that would support the HP. Our results show that such signal costs are neither sufficient nor necessary to explain honesty; *they are simply irrelevant for signal honesty*. Cost-free or even beneficial honest equilibria are possible: high-quality offspring need not waste energy to produce honest begging calls and peacock trains need not be wasteful or even costly to signal male quality. Honesty is maintained by the *potential costs* of cheating through dishonest signals, but not by some general cost of signalling at the evolutionary equilibrium (see [8, 31]). It follows that efforts to measure signalling cost at the equilibrium are not informative about honesty or stability of the signalling system. Similar arguments have previously been made [8, 31], and now our equations provide the first general mathematical proof.

Traditional explanations of honesty differentiate between signal costs (i.e. ‘handicaps’) and constraints (i.e. ‘indices’). Recent theoretical models seem to undermine such a dichotomy. First, these models suggest a continuum from cost-free cues to costly signals (see [26]), and second, they claim ‘that costly signalling theory provides the ultimate, adaptive rationale for honest signalling, whereas the index hypothesis describes one proximate (and potentially very general) mechanism for achieving honesty.’ (see [24], Abstract) While we agree that physical constraints pose a potential proximate mechanism to explain honesty, our results do not support the first part of the claim, namely that ‘costly signalling theory provides the ultimate, adaptive explanation for honest signalling...’ [24]. While we also agree with the claim that there is a continuum from cost-free to the most costly signals, there is also a potential continuum from cost-free to beneficial signals, which was ignored by costly signalling theory.

In summary, previous costly signalling models have used a set of unnecessarily restricted possible solutions to explain the evolution of honesty. Consequently, the costly signalling solutions provided by traditional costly signalling models are neither unexpected nor interesting, as the solutions are merely a consequence of the assumptions postulated by these models. Due to their restricted design, these models can only investigate costly signalling solutions because other solutions (e.g. cost-free or beneficial) are impossible within the boundaries of their additional *costly signalling model assumptions*. Given their restrictive set of assumptions, these models cannot provide general results for honest signalling or general predictions about the evolution of signals (Fig. 5). Our method provides a general calculation of signalling equilibria, and therefore, it should help research on honest signalling theory to progress beyond the domain of restricted costly signalling models.

These results highlight the need for a better framework than the erroneous HP (and costly signalling) for explaining honest signalling (see Fig. 5). Signals are expected to confer fitness trade-offs -*signalling trade-offs-*, which are better understood as life-history trade-offs rather than as ‘handicaps’, i.e. signals that are honest because they are costly. The seminal models that attempted to test the HP relied on life-history trade-offs to create differential costs between signallers that differ in quality: a trade-off between reproduction and survival in Grafen’s model [1] or a trade-off between current and future offspring in Godfray’s model [2]. A recent laboratory experiment shows the importance of condition-dependent trade-offs versus equilibrium costs of signalling [62]. A recent model that investigated the differences between additive and multiplicative fitness functions also adopts an evolutionary trade-off framework [29] (see Appendix 7 for differences between this and our approach). Our results here show how honesty can be selectively maintained by condition-dependent signalling trade-offs. Such trade-offs can be difficult to measure [63, 64], but this approach allows the use of theoretical models and empirical methodology established in this field [64–67].

Finally, there are several additional reasons for adopting the term ‘trade-off function’ instead of ‘cost function’ in signalling theory, as we propose here. The term ‘costly signalling’ has different meanings in the relevant disciplines (see Fig. 5). In economics, ‘costly signalling’ refers to models that apply a utility function with a cost term. In biology, ‘costly signalling’ is usually associated with the HP, and these terms are often used interchangeably. This semantic difference has contributed to confusing costly signalling theory with the HP, and given the misleading impression that the HP is supported by mathematical models [7]. This confusion is reason enough to avoid this term in biology. Moreover, the name ‘cost function’ in economics can be misleading when applied to biological signalling games. As we have seen, this function, regardless of its label, provides a *transformation* that does not have to realize an absolute cost, as we have seen in our case of the trade-off function *T*. In fact, *most biological models lack an explicit cost function*. Furthermore, cost-bearing utility functions in economics are usually additive, whereas fitness components in biology are generally multiplicative. In the additive case, one function can always represent a cost in the absolute sense. However, in the multiplicative case, this is not possible, as we have shown. In biology, *the trade-off is between benefit functions*. *There is no cost function* in the seminal models of mate choice [1] and parent-offspring signalling communication [2, 14, 15]: neither survival nor reproduction can be considered to be the costs. Labelling these models ‘costly signalling’ *sensu* economics is just as misleading as *sensu* biology. Thus, for these reasons, we suggest using ‘trade-off’ instead of ‘cost’ function, and indeed, *this term better reflects the key insight of biological signalling games*.

## Conclusions

Our results help to understand why the handicap paradigm needs to be rejected, and why signals — their costs, benefits, and trade-offs — are better understood using a Darwinian perspective (and conventional tools such as evolutionary game theory, optimality models, and life-history theory). Signals need not be costly or wasteful to be honest, and cost-free or even beneficial honest equilibria can be evolutionarily stable. Rather than being wasteful, *we should expect signals to be efficient*. Signals can be both honest and efficient, and cheating may prove to be less efficient (more costly and even wasteful) than honesty. They can be better understood from a Darwinian ‘Efficiency Principle’ [32] rather than from the erroneous Handicap Principle. Seemingly exaggerated signals, such as the peacock's train and deer antlers, might seem wasteful but they may provide minimal or no fitness cost to the bearer [61] — as honest signallers may produce them more efficiently than cheaters. There is no reason to suspect that signals evolve under a separate, non-Darwinian process of selection, contrary to Zahavi [4]; and since they evolve through natural selection like other traits, they should be efficient rather than wasteful.

## Methods

### The model

The model consists of two agents, the signaller **S** and the receiver **R**. The signaller elicits a signal to request an amount *a* of the resource from the receiver. The signaller’s fitness *w*_**S**_ depends on the signaller’s quality *q*, on the intensity of its signal (asking for an amount *a* of the resource) and on the amount of resource *z* provided by the receiver due to the signal. The receiver’s fitness *w*_**R**_ depends on the hidden quality *q* of the signaller and on its own response strategy, that specifies the amount of resource *z* the receiver shares with the signaller; $\hat{z}$ denotes the equilibrium amount (in honest equilibrium, it is expected that $\hat{z}=z=a$). The response can be written as a function directly dependent on *q*. We treat the signaller fitness *w*_**S**_ as an additive or multiplicative combination of a benefit function *B* and a signal trade-off function *T*, where both *B*(*q*, *z*) and *T*(*q*, *z*) are functions of signaller quality *q* and the received resource *z*. *T* defines the trade-off of asking for *z* = *a* amount of resource as a signaller of quality *q*, depending entirely on the signaller. *B*, on the other hand, is controlled entirely by the receiver’s response (how much *z* the receiver shares, based indirectly, through a signal, on the signaller’s quality *q*; see Appendix 1 for a formal derivation). This interpretation justifies the mathematical decomposition of *w*_**S**_ into these two functions. Derivatives are with respect to *z*; a hat over a symbol indicates equilibrium value. Table 1 lists the quantities of the model.

**Table 1:**
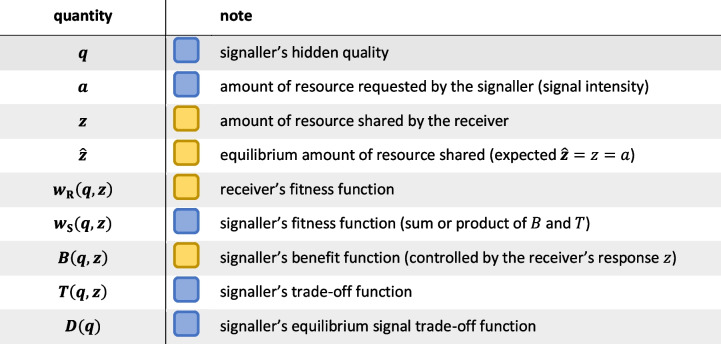
Notation used in the model. Coloured boxes indicate the controlling party, blue for signaller, yellow for receiver. For more details, see Appendix 1 Table S2

### Conditions of honest equilibrium

The honest signalling equilibrium has two conditions (for details, see Appendix 1):

1. *Condition of honest optimum* specifies that there exists an optimum amount of resource $\hat{z}$ that the receiver is willing to share. That is, the receiver, depending on the received signal, shares an amount that equals to the amount it would share if she could directly assess the signaller’s quality. This means, that signals are honest as they reveal the signaller’s quality, so that resource allocation is optimal for the receiver.
2. *Condition of shared interest* specifies that there is no conflict between receiver and signaller as the signaller asks the exact amount the receiver is willing to share and both *w*_**S**_ *and w*_**R**_ have their respective maxima at $\hat{z}=z=a$. Since neither the receiver nor signaller benefits by deviating from it, the condition implies stability. It has two conditions:
  - *Extremum condition* specifies that *w*_**S**_ has an extremum at $z=\hat{z}$:

1a $${w_{\textbf{S}}}^{\prime}\left(z=\hat{z}\right)=0.$$

2.b. *Stability condition* specifies that the extremum at $z=\hat{z}$ is a maximum:

1b $${w_{\textbf{S}}}^{\prime \prime}\left(z=\hat{z}\right)<0.$$

### Reverse-engineering the trade-off function

Finding honest signalling solutions in signalling games requires: (*i*) calculating the optimal resource sharing decision for the receiver, (*ii*) calculating signalling trade-offs (*T*, traditionally called a ‘cost function’) that transform the signaller’s optimal decision to the receiver’s optimum. That is, the signaller has optimal fitness when it asks for and receives the same amount *z* that the receiver is willing to share in its fitness optimum (see Fig. 1). We provide a formal method to reverse engineer the trade-off function *T* that is general and specifies all the infinite number of solutions. We use a Taylor series expansion of the signaller’s fitness *w*_**S**_ to specify the conditions of honest signalling identified by previous models [14, 22]. Since *w*_**S**_ is composed of *B* and *T* (additively or multiplicatively), *w*_**S**_ can be expressed as the appropriate combination of terms of the Taylor series of *B* and *T* (see Fig. 3 and Fig. S1). The first and second order Taylor coefficients of *w*_**S**_ can be used to express the stability and honesty conditions (Eqs. 1a, b) as constraints on the first and second derivatives of *B* and *T*. Since *B* is given, we use these constraints to construct a general trade-off function *T* that, when combined with *B*, yields a signaller fitness function *w*_**S**_ that fulfils the conditions of honest signalling (its optimum coincides with the optimum of *w*_**R**_). In Appendix 4, we apply our method to known models.

### Additive fitness model

First, we derive the general trade-off function for the additive model. For a visual guide, see the left panel of Fig. S1, for details, see Appendix 2. In the case of the additive fitness model, the signaller’s fitness is the sum of the benefit and trade-off functions:

2 $${w}_{\textbf{S}}=B+T.$$

Both *B* and *T* can be written as Taylor series around the equilibrium $z=\hat{z}$ (omitting function arguments *q* and *z* for sake of simplicity):

3 $$B(z)=B\left(\hat{z}\right)+\frac{B^{\prime}\left(\hat{z}\right)}{1!}\left(z-\hat{z}\right)+\frac{B^{\prime \prime}\left(\hat{z}\right)}{2!}{\left(z-\hat{z}\right)}^2+\dots,$$

4 $$T(z)=T\left(\hat{z}\right)+\frac{T^{\prime}\left(\hat{z}\right)}{1!}\left(z-\hat{z}\right)+\frac{T^{\prime \prime}\left(\hat{z}\right)}{2!}{\left(z-\hat{z}\right)}^2+\dots .$$

With *β*_*k*_ = *B*^(*k*)^/*k*! and *τ*_*k*_ = *T*^(*k*)^/*k*!, the sum of *B* and *T* can be rewritten:

5 $${w}_{\textbf{S}}\left(q,z\right)=\left({\beta}_0+{\tau}_0\right)+\left({\beta}_1+{\tau}_1\right)\left(z-\hat{z}\right)+\left({\beta}_2+{\tau}_2\right){\left(z-\hat{z}\right)}^2+\dots .$$

At the equilibrium $z=\hat{z}$, conditions Eqs. 1a, b must be met by *w*_**S**_. According to Eq. 1a, the first derivative of *w*_**S**_ must be zero:

$${w}_{\textbf{S}}^{\prime }=0,$$

$${\beta}_1+{\tau}_1=0,$$

$${\tau}_1=-{\beta}_1,$$

$${\tau}_1=-{B}^{\prime }.$$

According to Eq. 1b, the second derivative of *w*_**S**_ must be smaller than zero:

$${w}_{\textbf{S}}^{\prime \prime }<0,$$

$${\beta}_2+{\tau}_2<0,$$

$${\tau}_2<-{\beta}_2,$$

$${\tau}_2<-\frac{1}{2}{B}^{\prime \prime }.$$

The inequality is always satisfied if *ε* > 0:

6 $${\tau}_2=-\frac{1}{2}{B}^{\prime \prime }-\varepsilon .$$

Substituting *τ*_1_ and *τ*_2_ into Eq. 4 and *D*(*q*) for $T\left(q,\hat{z}\right)$, the constructed trade-off function for additive fitness components is:

7 $$T\left(q,z\right)=D(q)-{B}^{\prime}\left(z-\hat{z}\right)-\left(\frac{1}{2}{B}^{\prime \prime }+\varepsilon \right){\left(z-\hat{z}\right)}^2+\dots .$$

### Multiplicative fitness model

In the case of the multiplicative fitness model, the signaller’s fitness is the product of the benefit and trade-off functions (for a visual guide, see the right panel of Fig. S1, for details, see Appendix 3):

$${w}_{\textbf{S}}=B\cdot T.$$

The Taylor series of a multiplicative *w*_**S**_ is the product of the individual Taylor series of the composite functions *B* and *T* (Eqs. 3, 4):

$${w}_{\textbf{S}}\left(q,z\right)={\beta}_0{\tau}_0+\left({\beta}_0{\tau}_1+{\beta}_1{\tau}_0\right)\left(z-\hat{z}\right)+\left({\beta}_0{\tau}_2+{\beta}_1{\tau}_1+{\beta}_2{\tau}_0\right){\left(z-\hat{z}\right)}^2+\dots$$

At the equilibrium $z=\hat{z}$, conditions Eqs. 1a, b must be met by *w*_**S**_. According to Eq. 1a, the first derivative of *w*_**S**_ must be zero (omitting function arguments):

$${w}_{\textbf{S}}^{\prime }=0,$$

$${\beta}_0{\tau}_1+{\beta}_1{\tau}_0=0,$$

$${\tau}_1=-\frac{\beta_1{\tau}_0}{\beta_0},$$

$${\tau}_1=-\frac{B^{\prime }T}{B}.$$

The first derivative of *T* at the equilibrium depends on *T* itself, unlike in the additive case. According to Eq. 1b, the second derivative of *w*_**S**_ must be smaller than zero (substituting *τ*_1_ from above):

$${w}_{\textbf{S}}<0$$

$${\beta}_0{\tau}_2+{\beta}_1{\tau}_1+{\beta}_2{\tau}_0<0,$$

$${\tau}_2<-\frac{\beta_1{\tau}_1+{\beta}_2{\tau}_0}{\beta_0},$$

$${\tau}_2<-\frac{\tau_0\ }{\beta_0}\left({\beta}_2-\frac{{\beta_1}^2}{\beta_0}\right).$$

The inequality is always satisfied if *ε* > 0:

$${\tau}_2=-\frac{\tau_0\ }{\beta_0}\left({\beta}_2-\frac{{\beta_1}^2}{\beta_0}\right)-\varepsilon,$$

$${\tau}_2=-\frac{T}{B}\left(\frac{B^{\prime \prime }}{2}-\frac{{\left({B}^{\prime}\right)}^2}{B}\right)-\varepsilon .$$

Substituting *τ*_1_ and *τ*_2_ into Eq. 4 and *D*(*q*) for $T\left(q,\hat{z}\right)$, the constructed trade-off function for multiplicative fitness components is:

$$T\left(q,z\right)=D(q)-\frac{B^{\prime }D(q)}{B}\left(z-\hat{z}\right)-\left(\frac{D(q)}{B}\left(\frac{B^{\prime \prime }}{2}-\frac{{\left({B}^{\prime}\right)}^2}{B}\right)+\varepsilon \right){\left(z-\hat{z}\right)}^2+\dots .$$

## Supplementary Information

**Additional file 1: Appendices 1-7**. **Figs S1-S8**, **Tables S1-S3**. **Appendix 1.** Conditions of honest signalling; **Appendix 2.** Derivation of the general trade-off function for the additive model; **Appendix 3.** Derivation of the general trade-off function for the multiplicative model; **Appendix 4.** Trade-off functions of well-known models; **Appendix 5.** Single step optimization models of signalling; **Appendix 6.** The model of (Biernaskie et al. 2018); **Appendix 7.** The model of [29].

**Additional file 2.**

## Data Availability

All data and models are available in the main text and in the Additional files.

## Abbreviations

HP: Handicap Principle
