# Supplementary figures and images for: Honesty in signalling games is maintained by trade-offs rather than costs
Source: BMC Biol. 2023 Jan 8;21:4. doi: 10.1186/s12915-022-01496-9 (PMC9827650; doi:10.1186/s12915-022-01496-9)

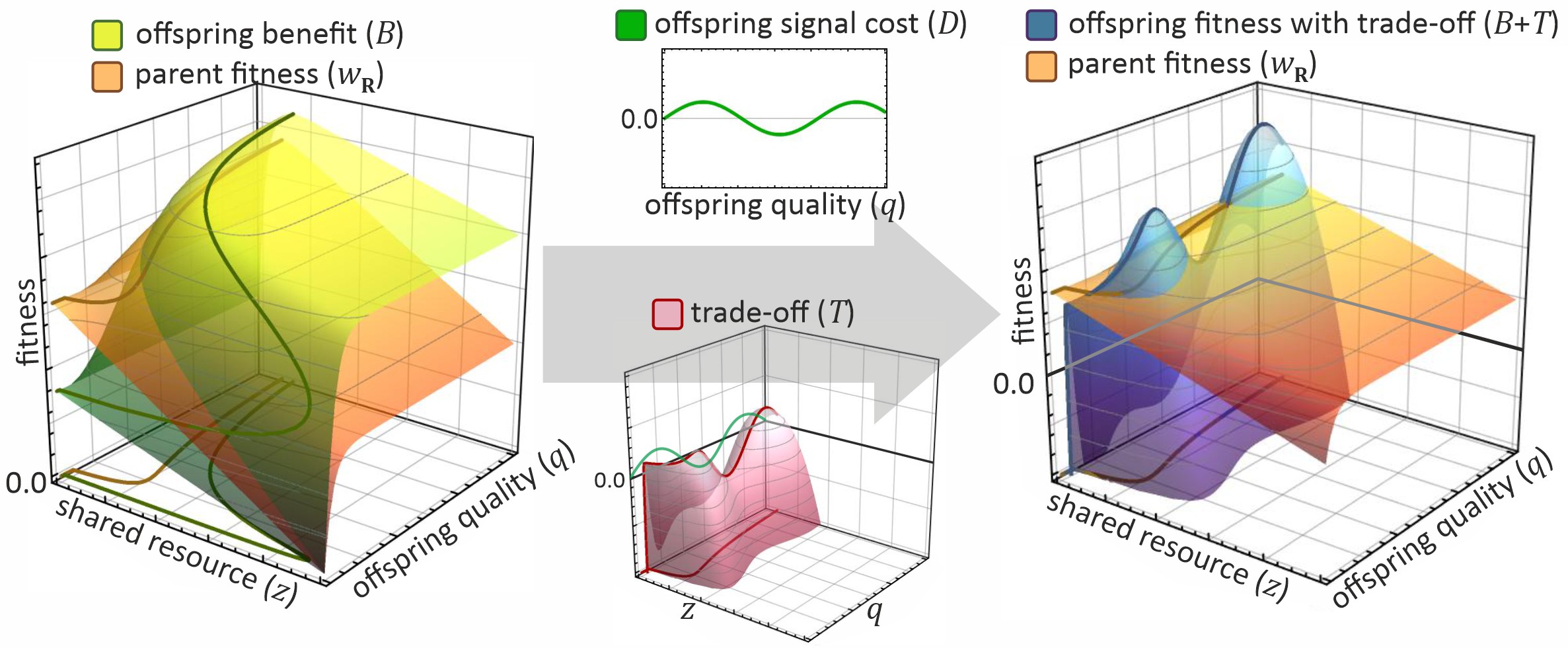

Supplement: Supplementary file 2 — Additional file 2. [file 12915_2022_1496_MOESM2_ESM.jpg]
